# Preparing effective medical illustrations for publication (Part 2): software processing, drawing and illustration

**DOI:** 10.2349/biij.4.2.e12

**Published:** 2008-04-01

**Authors:** SC Wang

**Affiliations:** Department of Diagnostic Radiology, Yong Loo Lin School of Medicine, National University of Singapore, Singapore

## INTRODUCTION

This is the second part of an article on preparing images for medical publication. The first part dealt with optimal capture and export of pixel-based medical images. This part will deal with post-processing and editing of such images using specific software tools, and with the use of graphics illustration and charting software applications for creation of medical illustrations and charts.

In the last 20 years, increasingly powerful computer software and hardware has become available to the general public, and the now-pervasive ability to create digital images through electronic radiology image archives, digital photography, scanning of paper or film and graphics software for charting and illustration, have meant that the once-arcane art of graphic design and medical illustration have become democratised and at least for many types of illustration, is no longer the province of the expert graphic artist. This is not to say that we can all become professional artists, but rather that the simpler tasks of medical illustration are now within the reach of the ordinary person with sufficient knowledge, tools, training and practice.

This article highlights and describes important approaches to producing high quality medical illustrations for publication, which has differing requirements to electronic, web-based or computer presentations. As such, it will cover general preparation, the types of illustration needed, key software that should be used, and some basic concepts and techniques that anyone preparing their own images should be familiar with.

## PRINCIPLES

If there is a key principle to be remembered by would-be authors, it is this: the published illustration *should be able to stand alone*. In short, if the illustration cannot be understood in the absence of any explanation other than the accompanying caption, it is incomplete. Thus, a chart usually needs a title and/or subtitle, legend and sometimes data points to be highlighted. A medical image or set of images often need alphanumeric identification and appropriate arrows or other symbols highlighting specific features. And drawings usually require labels attached to various elements.

Furthermore, simplicity is crucial. It is very important for anyone in the target audience to be able to rapidly grasp what the illustration is meant to show, ideally *without* reading the caption (this is particularly true for charts and tables). Complex graphics and illustrations will generally not be understood without intensive study, unless there is appropriate division of information presentation. For many charts in particular, careful selection of the type of graphic for the data is important to ensure that the presentation is not only meaningful but avoids distracting and sometimes misleading graphic “embroidery” which is so easily applied using current charting packages. For scientific applications, dedicated scientific charting packages are to be preferred to business-style charts available in spreadsheet software such as Microsoft Excel.

## ILLUSTRATION TYPES

Today, all illustrations are digital in nature, or become so in the course of publication preparation, simply because publishing technology is now universally digital, with all journals now laid out using computers and dedicated professional software tools. There are two types of graphics used for illustration: pixel-based images and vector graphics. Typically all such publications require that digital graphics are submitted in a finalised form suitable for placement, with specific resolutions and file formats which can be used directly for page layout. Regardless of the original type of image, the submitted images for publication are typically pixel-based image files (usually tagged image file format, or TIFF) at the resolution required by the publisher. The majority of images for eventual publication will have to be exported to this format, as it is rarely used for primary image capture or illustration creation. However, its universality in all imaging software and innately lossless image storage format means it is ideal for the final common step prior to publication.

Pixel-based graphic acquisition and capture was dealt with in Part 1 of this 2-part article. Even when captured from the original source, these images usually require re-sizing to a specific pixel resolution and size, grey-scale manipulation, and almost always need some on-image indicators and annotation (alphanumeric characters, asterisks, arrows etc.) prior to export for publication. Typically software such as Adobe Photoshop is used for this purpose. Some basic rules should be followed to minimise any loss of quality during image manipulation with such software; these guidelines are described below.

Vector images differ substantially from pixel-based images. In digital form they are resolution-independent, and can be scaled almost infinitely in size without any reduction in quality. They are created using mathematically defined primitive objects such as lines, arcs, rectangles and bezier curves, with mathematically defined shading, line width, colours etc, by either charting packages such as DeltaGraph or Kaleidagraph, or graphic illustration packages such as Adobe Illustrator. Because these packages have proprietary formats, the images must be exported to common pixel-based formats prior to publication; the author must render them to a specific size and pixel resolution suitable for printing. Typically this is done by one of 3 methods:

- Exporting to Adobe Portable Document Format (PDF) to a specified size; this usually requires the purchase of Adobe Acrobat.
- Export directly to Tagged Image File Format (TIFF) to the specified resolution; this is usually possible without additional cost
- Export to Adobe Encapsulated Postscript (EPS), with subsequent rendering of the image using Adobe Photoshop by the publisher to the resolution required; again this usually does not require any additional cost

The last option gives the author and the publisher the most flexibility, but is rarely used except by professional illustrators.

### Software Considerations

The ubiquity and power of Adobe’s Photoshop software for image editing has led to a justifiably legendary reputation. However, the full version of Photoshop is overkill for the vast majority of tasks the radiologist needs to create useful images for presentation or publication. There are some much cheaper alternatives for the more casual user, which have the benefit of a shorter learning curve and sometimes much simpler methods of performing common correction tasks.

The best of these is probably Adobe’s much cheaper Photoshop Elements, which for routine tasks is the equal of the full product. Free or low cost shareware alternatives are also widely available on the internet; GIMP (the GNU Image Manipulation Program) is a well known example of very powerful, open-source, free image manipulation software. However, this is actually somewhat more complex to learn than Photoshop.

A major advantage of the Photoshop family of products, including Photoshop Elements, is the ability to use a huge range of third party plug-in software tools to facilitate image correction. These include software for RAW format conversion, for removing or reducing image noise, for correcting lens distortion in digital photographs, and for more accurate interpolation when enlarging an image (Photoshop’s internal algorithms show significant artifacts for enlargement factors of 200% or greater).

### Adjusting Images

This is required in virtually every medical image that is captured, regardless of how it was obtained. The exposure, brightness and contrast may adjusted for the screen but not be optimal for printing. There may be image distortion or noise. The image may be too large or too small and require scaling and resizing. Text on the image (e.g., patient name and ID, examination details) or artifacts such as greaseproof pencil marks, scratches and reflections may need to be removed (particularly so with images photographed from hardcopy film printing) or appropriate markers and text inserted (e.g., arrows, stars and alphanumeric indicators of specific features). One universal step for publication is the need for image sharpening; unless this is done, even the best images tend to be reproduced appearing slight “soft” and blurred due to the screen and printing process.

**Figure 1:**
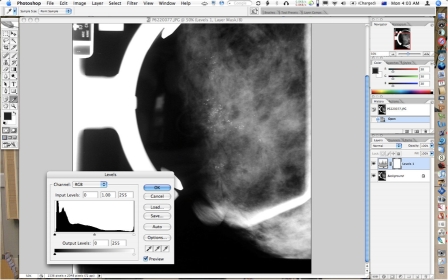
Screenshot of Photoshop with Levels adjustment layer added and dialog opened for adjustment of image brightness and contrast as well as greyscale range.

**Figure 2:**
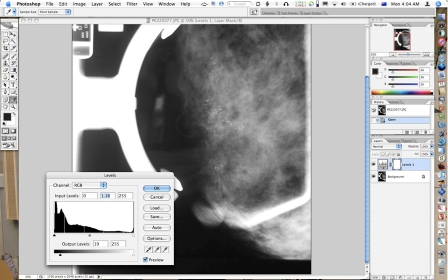
Image preview after levels adjustment performed.

**Figure 3:**
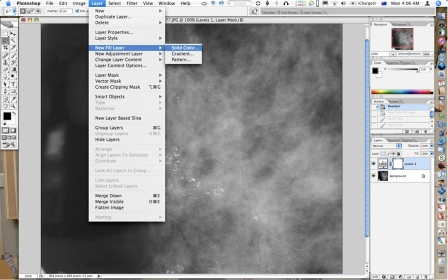
After cropping, creating a Fill Layer with a solid dark grey fill.

**Figure 4:**
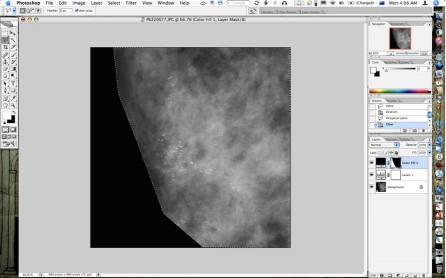
Fill layer edited roughly with the lasso tool to reveal most of the desired underlying image; this will be touched up with the brush tool.

**Figure 5:**
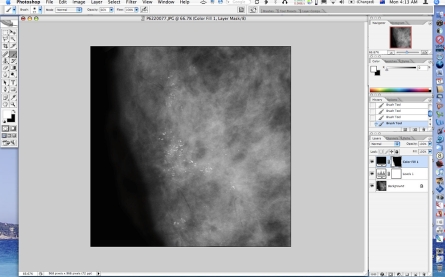
Image after fill edge editing. Greasepoint pencil marks which were visible were removed using the healing brush tool.

**Figure 6:**
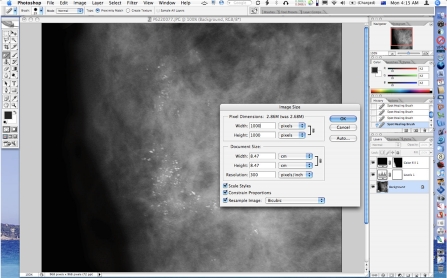
The image is then resized and sharpened.

**Figure 7:**
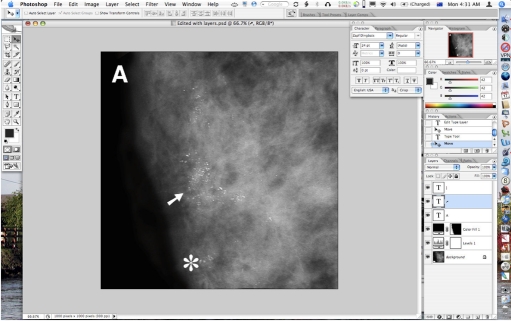
Text, arrows and stars are added using the Text tool; this creates a new layer for each text element which remains editable and repositionable at all times. This is saved as a Photoshop (.PSD) file with all layers intact.

**Figure 8:**
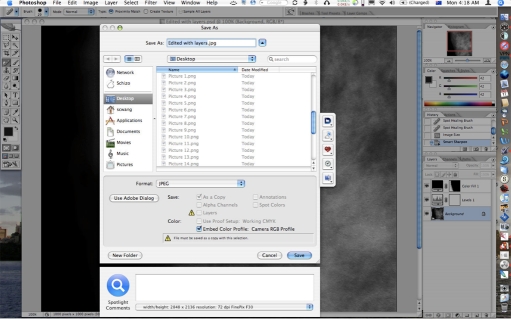
Saving the image as a JPEG or single layer TIFF file collapses all the layers into a single flattened image for publication.

Virtually every images must undergo multiple editing steps, leaving any resizing and sharpening effects to the last as these are destructive, and if done too early cause major artefacts subsequently (see Figures 1 to 8). Specific tool terminology used here refers to Adobe Photoshop naming conventions unless otherwise stated.

**Figure 9:**
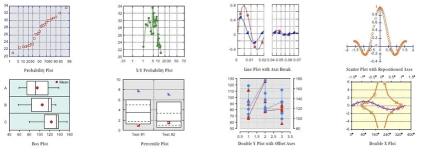
Examples of various charts created by scientific charting software.

- Correcting lens distortions (e.g., LensFix)
- Levels adjustment for overall brightness and contrast
- Cropping or masking image to desired region
- Editing small regions to remove artifacts using the brush, stamp and healing brush tools
- Enhancing various features using lasso and mask selection and various filters
- Resizing to final desired size; this may require special software tools to ensure minimum loss of quality (e.g., pixl Smartscale, PhotoZoom Pro)
- Adding text, stars or arrows as required
- Sharpening, using Unsharp Mask or similar tool
- Saving file with layers in native or multilayer TIFF format for redo editing
- Saving or exporting to TIFF without layers for publication

For maximal flexibility, most modern pixel editing software support the use of layers to perform these editing tasks. This is nondestructive to the underlying original image, permits re-do editing at any time, and can be used to create a “flattened” final image for publication once all final adjustments have been made. It is well worth learning to use this feature for many image editing applications.

## ANNOTATING IMAGES

Most images for publication in a medical journal require some text, arrows and other indicators to be overlaid on the image to highlight specific features for discussion.

In the past, adhesive precut lettering and symbols (e.g., Letraset) would be rubbed onto a camera-ready photograph by the author prior to sending the article for review.

As noted above, it is possible to use Photoshop to create layers of text and symbols to overlay the image, and to send this to the publisher. In general, although it is possible to flatten the file as described above, it is usually important to try to use resolution independent text rather than to fix the resolution of the text with the image file.

Typically this issue can be problematic for both the author and the publisher. However, the ubiquity of Microsoft Office’s Powerpoint software application has recently led some publishers to request that all publication images to be sent in the form of Powerpoint files, with the images, drawings and charts embedded on separate slides and any text, arrows or other symbols simply overlain on them using Powerpoint’s built-in drawing tools, which are easy to use and quite suitable for this task. Any annotations can be made using Powerpoint’s built-in notes feature. In this fashion, the inbuilt resolution of the image can be retained (the publisher can copy and paste the image readily into a photo editing package such as Photoshop) and the resolution-independent text, arrows etc can be accurately positioned so the publisher can see exactly what the author intended.

Ultimately the would-be author must supply the images in a well-organised, appropriate quality fashion as requested by the publisher’s instructions to authors.

## VECTOR-BASED IMAGE SOFTWARE

Although medical images are universally resolution-dependent and pixel-based, virtually all charting and graphing software produces resolution-independent images that are vector-based; i.e., the graphic file represents a series of instructions to draw graphic primitives using precise mathematical descriptors rather than pixel by pixel descriptions of an image. These images can be resized to any resolution desired prior to final export and printing.

There are three major types of such software:

- Software designed to automatically create a wide range of charts and graphs from data that has been entered into a table or database (e.g., Excel, Kaleidagraph, Deltagraph),
- Software designed to permit drawing a relatively limited range of predetermined shapes and objects using graphic libraries to create diagrams (e.g., Visio, Omnigraffle, Powerpoint)
- Software designed to draw virtually any shape or object using highly sophisticated drawing tools (e.g., Adobe Illustrator, Corel Draw, Macromedia FreeHand).

### Charting Software

Most users are familiar with the built-in charting tools in Microsoft Office, which create usable business charts readily, but which lack most of the tools needed to create sophisticated scientific charts and graphs (e.g., box plots, whisker plots, automatically calculated error bars, splines, regression lines, curves of best fit etc.). Each software programme usually requires considerable effort to master, but if you create such charts frequently this effort is well worthwhile.

For publication, a few simple rules for such charts should be followed, namely:

- Always ask yourself what exactly you are trying to show with the chart. This should ideally be with one or two clear unambiguous sentences. If some data elements do not fit this explanation, they should probably be omitted from the chart.
- 2-dimensional charts are the rule; 3-dimensional charts are difficult to read accurately and to obtain useful comparisons between datasets. “Pseudo-3D” effects in particular are meaningless.
- All charts should be simple black and white line drawings for printing, with minimal greyscale shading, patterning, series marking and other effects; this should be kept only to what is absolutely necessary to show the differences between datasets. Colour can be sparingly and effectively used for onscreen presentations and web publication.
- Continuous data can be represented by a line connecting data points; discontinuous data should either be presented as a scatter plot or as a histogram or column chart
- Try to have no more than 6 datasets represented on a single chart - having any more makes the chart very difficult to interpret; large numbers of datasets can be represented either by aggregated data or a series of charts representing subsets of the data
- Each chart should have a title, and each axis should be labelled clearly including the units of measure and data categories as appropriate. A legend is desirable if there are multiple datasets
- Keep gridlines, chart shading and text datapoints to a minimum
- Avoid duplicating the data in a chart in an accompanying table, unless the chart is to show a very specific trend or feature of the data that is difficult to extract from the table.
- Always preview and adjust the chart at the expected final size for printing; it is surprising how often such “automatic” charting changes the relationship and position of elements between different sizes and shapes of the various elements of the chart (Excel is particularly prone to this)

### Primitives, Freeforms & Beziers

People familiar with the Drawing environment of Microsoft Office are familiar with the concept of drawing with predefined primitives such as ovals, rectangles, polygons, arcs, lines and arrows. These greatly simplify the process of drawing many simple diagrams, and with more sophisticated tools such as Microsoft Visio or Omnigraffle, can create quite complex diagrams.

**Figure 10:**
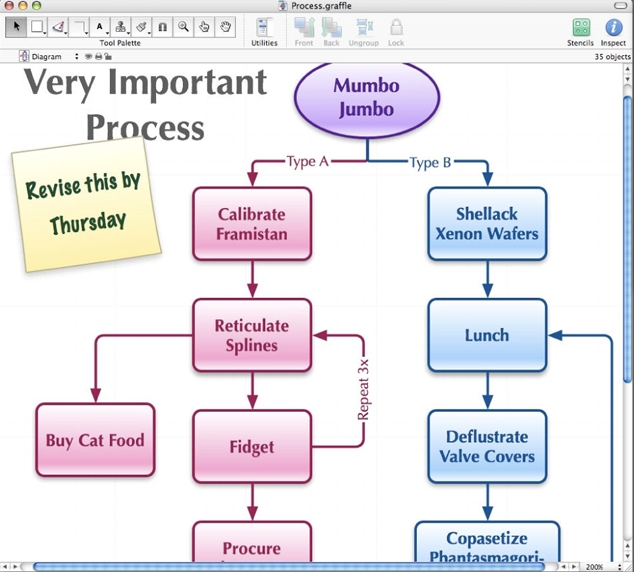
A simple workflow diagram created using object-oriented drawing tools such as those found in Microsoft Office, Microsoft Visio or OmniGraffle.

This type of software is quite useful for many applications in teaching of Radiology, including process flow charts, clinical decision algorithms, Venn diagrams, physics principles and so on (Figure 10). The simple tools within Microsoft Office are quite limited, so any complex diagrams probably require additional purchase and learning a further application.

### Professional Drawing Applications

The software tools described above are not suited to creating complex drawings of objects with numerous layers, photorealistic rendering or extremely finely detailed graphics; such tasks require professional level illustration software such as Adobe Illustrator, and are again overkill for most Radiology illustrations for publication. In general, most scientific users and radiologists will have little need for this software; the complexity of learning and using these tools effectively is too rarely used and is best left to graphics professionals.

**Figure 11:**
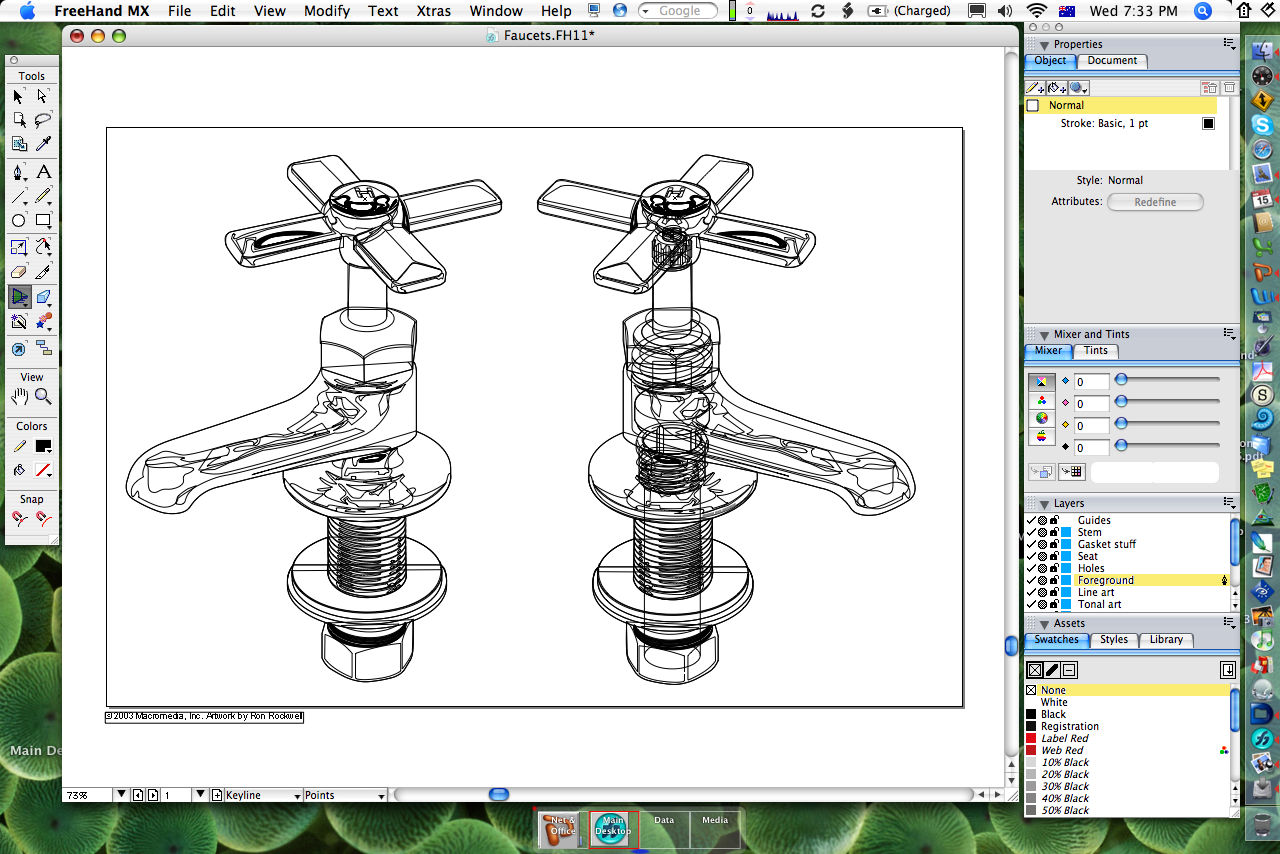
Vector bezier-type drawing software (Macromedia FreeHand) showing the “wireframe” line depiction of a drawing of two taps, with all shading and colour rendering off.

**Figure 12:**
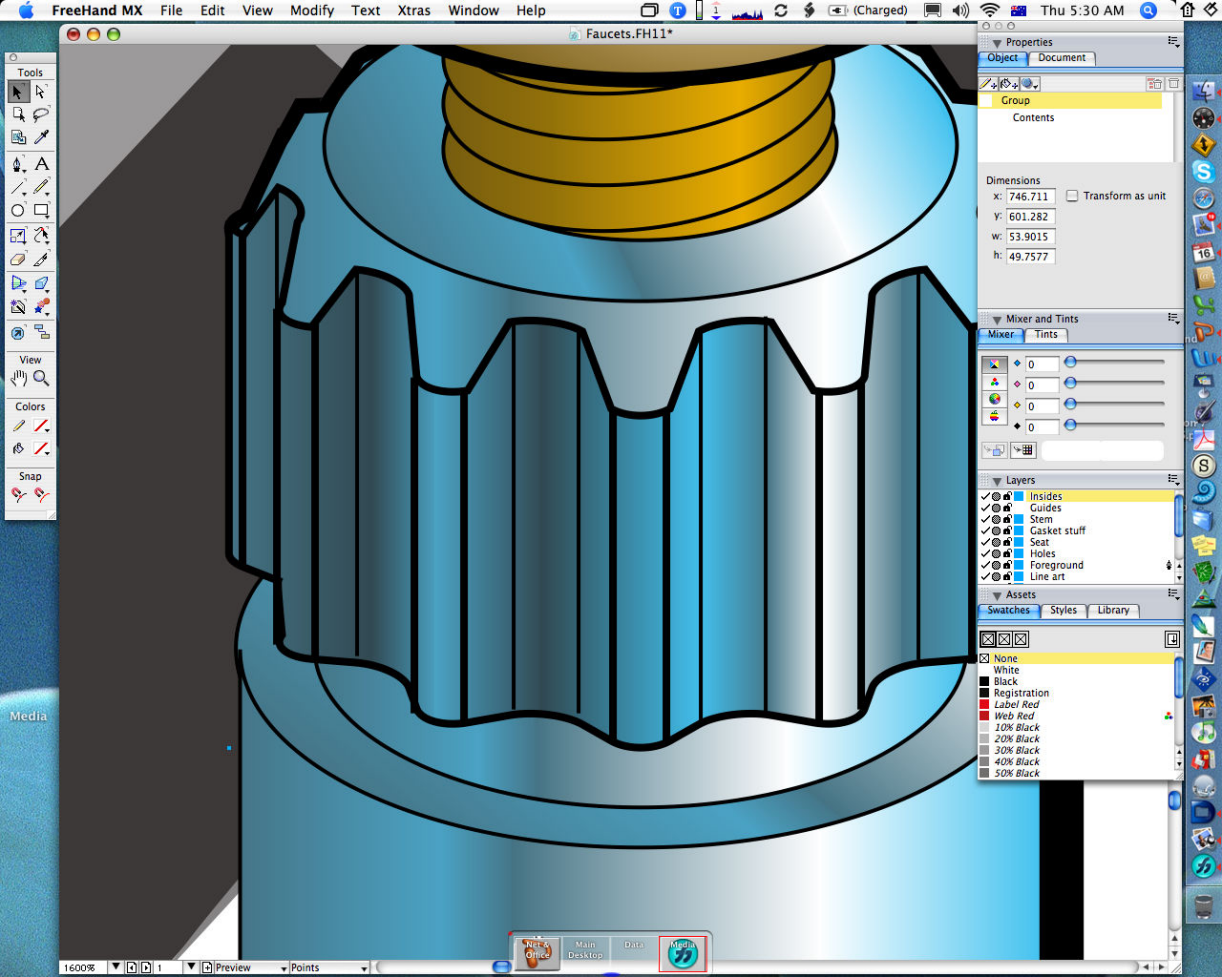
1600% magnification view of a portion of the same drawing with shading and colour rendering on. Note the preservation of fine detail and precision in the image.

These applications are sophisticated drawing environments and allow for images of almost infinite size to be created at extremely high levels of detail. They are ideal for creating structural drawings or graphics using complex effects (Figures 11 and 12).

## GET ORGANISED

All modern computer operating systems have built-in software for managing and viewing images stored in various folders in the user’s document filing system. However, this built-in software is not very useful for very large image collections, sophisticated searches, filing and archiving. In particular such software is unable to handle DICOM images.

**Figure 13:**
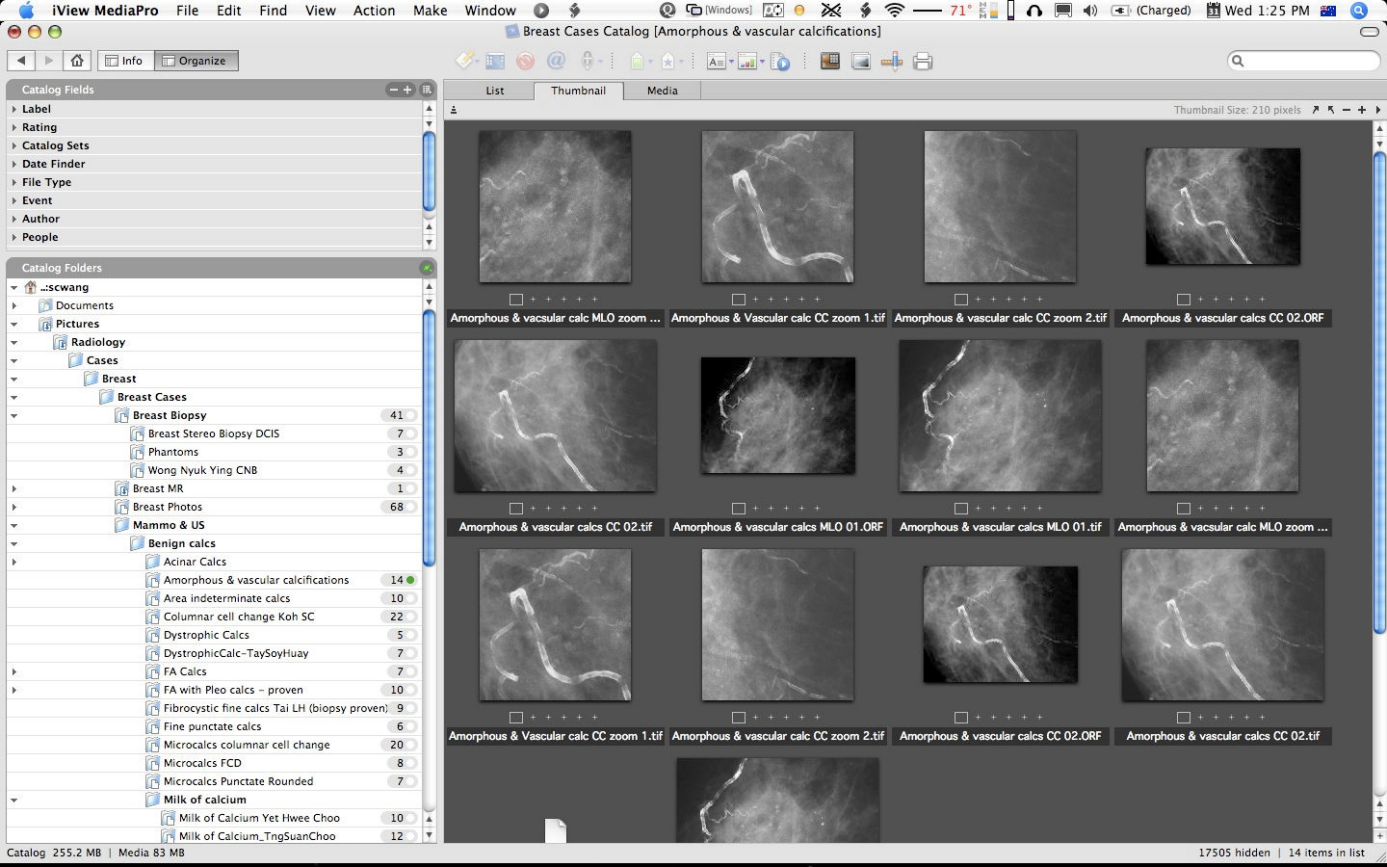
Screenshot of a typical image management software application showing multiple versions of images which can be organised heirarchically in folders as shown in the file browser on the left. The images can be sorted, renamed, even filed offline on external media, and opened with other applications for adjustment and editing.

A software tool which will become indispensable after more than a few dozen images are obtained is image database software (Figure 13). The most ubiquitous is probably the Adobe Bridge programme, as it is bundled with every copy of Photoshop and Photoshop Elements. Moreover, the latest Photoshop CS3 Extended Edition directly supports cataloguing, opening and editing DICOM image files without an intermediate conversion step.

There are several other powerful packages, which can maintain separate fully linked and indexed databases of images (even on external storage media) and which can also link between images of related type, subject, place, date etc. according to various flexible keyword categories assigned by the user. In addition, some applications also have powerful version control, inbuilt RAW image editing and processing tools, can export and repurpose images according to publication requirements as needed, and are eminently suited to a professional collection of thousands of high resolution images. Examples of such software applications include Adobe Lightroom, Apple Aperture, and Microsoft Expression Media.

Images should in general be organised by organ system, by pathology, by case and so on, and file naming and tagging should be performed fairly regularly to ensure that as much information as possible is available for future searches and utilisation.

The importance of backup of the images cannot be overemphasised, as hard disks are intrinsically unsafe methods of long term archival storage, being prone to sudden catastrophic hardware failure, particularly as they age. There is little worse for a radiologist than to realise the entire annotated carefully edited set of images of a particularly rare irreplaceable case, or even an entire collection of cases, has been lost forever due to hard drive failure.

Archival backup and storage of multiple copies of the images on high quality optical media, preferably one or two sets representing the original, untouched images, and one or two sets representing the edited, annotated image sets and their storage heirarchies. Backup should be performed regularly, ideally daily or at least weekly, with a regular systematic approach.

## CONCLUSIONS

There is more to creating good illustrations for publication than meet the eye. The paramount consideration should be whether the reader will find the illustrations informative and easily interpretable without resorting to reading the text. If the images fail this simple test, they are deficient and require additional thought, planning and redesign.

There is a plethora of software tools available that can easily create elegant clear graphics and illustrations which readily explain a scientific principle. Unfortunately this same software can be used just as easily to create complex, unclear, poorly presented information which could either obscure the main principles or confuse the reader. The difference lies in the end user and his/her use of the software. The availability of such tools does not remove the onus on the author to be clear, both in mind as to what the illustration is meant to show, as well as how to best depict the relevant information.

And as always, it is crucial for the author(s) to carefully read the instructions to authors, especially for print publications, which tend to limit printing to black and white/greyscale and which often state explicitly a limit to the number of illustrations and the specifics of image size and resolution as well as file formats required. Deviation from these guidelines may mean increased expense to the authors, or more commonly, nonpublication of images and charts one has laboured hard to create.

## RECOMMENDED SOFTWARE

### Pixel Editing

1. Adobe Photoshop Elements 6 (www.adobe.com)

2. Adobe Photoshop CS3 Extended Edition (www.adobe.com)

3. Neat Image (noise reduction) (www.neatimage.com)

4. LensFix & PanoTools (lens distortion correction) (www.kekus.com)

### Scientific Charting

5. Kaleidagraph (www.synergy.com)

6. Deltagraph (www.rockware.com)

### Diagramming

7. Omnigraffle (www.omnigroup.com)

8. Microsoft Office & Visio (www.microsoft.com/office/visio/)

### Drawing & Illustration

9. Adobe Illustrator (www.adobe.com)

### Image Cataloguing, Organisation and Workflow

10. Adobe Bridge (www.adobe.com)

11. Microsoft Expression Media (www.microsoft.com/Expression/)

12. Apple Aperture (www.apple.com)

13. Adobe Lightroom (www.adobe.com)
